# Consumer understanding, preferences and acceptance of front-of-pack labels in Thailand: foundational evidence for policy development

**DOI:** 10.1017/S1368980026101852

**Published:** 2026-01-22

**Authors:** Payao Phonsuk, Christine Johnson Curtis, Phasith Phatchana, Suladda Pongutta

**Affiliations:** 1 Department of Health Education and Behavioral Sciences, Faculty of Public Health, Mahidol University, Bangkok, Thailand; 2 Resolve to Save Lives, New York, NY, USA; 3 Siriraj Health Policy Unit (SiHP), Faculty of Medicine Siriraj Hospital, Mahidol University, Bangkok, Thailand; 4 International Health Policy Program Foundation, Nonthaburi, Thailand

**Keywords:** Front-of-pack labels, Nutrition policy, Consumer behaviour, Thailand, Food labelling

## Abstract

**Objective:**

This study aimed to assess the understanding, perceptions and preferences of different front-of-pack labelling (FOPL) formats among Thai consumers.

**Design:**

We conducted a mixed-methods study comprising a cross-sectional online survey and semi-structured interviews between February and March 2022. The survey assessed comprehension and preferences for six FOPL formats (Guideline Daily Amounts (GDA), Healthier Choice logo (HCL), warning labels (WLs), Nutri-Score, Health Star Rating and Traffic Light labels). Quantitative data were analysed using descriptive statistics, chi-square tests and multiple logistic regression. Qualitative data underwent thematic analysis.

**Setting:**

Bangkok and metropolitan areas

**Participants:**

Thai residents aged 12–78 years (*n* 410)

**Results:**

While awareness of existing labels was high (GDA: 95·4 %, HCL: 82·4 %), only 23·9 % regularly read GDA labels. WLs and Nutri-Score were the most effective at providing information to consumers in a format that translated into choosing healthier products. WLs demonstrated the highest effectiveness in guiding healthier choices. HCL received the highest agreement across multiple attributes, including packaging inclusion preference (59·8 %), visibility (58·5 %) and visual appeal (57·3 %), although effectiveness was not tested. Qualitative findings revealed preferences for colour-coded systems but identified barriers including time constraints, small font sizes and difficulty interpreting numerical information.

**Conclusions:**

While interpretive labels, particularly WLs, are most effective for guiding consumers to healthier choices, successful implementation requires consideration of both consumer preferences and real-world usage constraints. Findings support replacing the current GDA system with an interpretive design, accompanied by comprehensive public education campaigns. These results provide evidence-based recommendations for FOPL policy development in Thailand.

Diet is the leading cause of non-communicable diseases globally^(1)^. A key feature of the food environment is the increasing availability of packaged foods, which are often high in sugar, fat and/or saturated fat and are heavily promoted^(2)^. As countries confront increasing rates of non-communicable diseases, such as CVD, stroke and diabetes, there is wide interest in implementing effective nutrition policies^(3)^. Creating supportive food environments is a recommended and cost-effective strategy that supports industry reformulation and behaviour change^(4–6)^.

The WHO has endorsed front-of-pack labels (FOPL) on packaged food as one component of a comprehensive policy approach to improve dietary intake and as a highly effective and cost-efficient ‘best buy’ for preventing non-communicable diseases^(7,8)^. The information included on packaged food, including branding, design, characters and nutrition claims, is the main way that food manufacturers communicate with consumers^(9)^. Governments around the world typically regulate much of the information that appears on packages to varying extents, including the ingredient lists, nutrient declarations, nutrition and health claims and supplementary information, which includes FOPL. FOPL provides an opportunity to inform consumers about the healthfulness of the product at the point of decision-making and is now widely implemented, although the design varies considerably^(10)^.

WHO recommends that FOPL be ‘interpretive, based on symbols, colours, words and/or quantifiable elements’^(9)^. Interpretive systems provide easy references for consumers to understand the healthfulness of a product and may include a summary indicator, such as a grade or summary score. FOPL warning labels (WLs), which highlight when foods are high in certain nutrients of concern, are a type of interpretive system. Non-interpretive systems, such as the Guideline Daily Amount (GDA) label, provide nutrient content information but do not provide consumers with information on healthfulness^(11)^. Interpretive systems are more likely than non-interpretive systems to prompt industry reformulation^(10)^. WHO has established fifteen guiding principles for FOPL, which include that the design should be understandable to population subgroups, enable comparison between food categories and within a food category and be supported by a public education campaign^(9)^.

Thailand has established itself as a leader in putting in place nutrition policies, such as back-of-package nutrition labels, a mandatory and a voluntary FOPL and the elimination of industrial trans-fat from the food supply^(12,13)^. FOPL in Thailand was developed in the context of industry influence^(12,14)^. Thailand currently has two different FOPL: a mandatory GDA label^(12)^ and the voluntary Healthier Choice logo (HCL)^(15)^ (Figure 1 (a) and (f)) that companies can use to identify healthier options that meet the Healthier Choice nutritional criteria. As of 2023, approximately 11 % of all packaged food products display the HCL, and approximately 40 % of eligible products display the label; a recent study found that adoption remains inconsistent^(15)^. While the GDA label has been in place for more than 14 years, studies have shown limited consumer understanding of the GDA label. A national survey in 2021 found that only 24 % of Thai consumers could correctly interpret GDA label information, with lower comprehension rates among those with less education and lower income levels^(16)^. Research in urban Thailand revealed that while consumers showed high awareness of the GDA label, their understanding and use of these labels for purchasing decisions remained limited^(17)^. This gap between awareness and effective use has prompted Thai health authorities to explore more intuitive FOPL systems.

**Figure 1.**
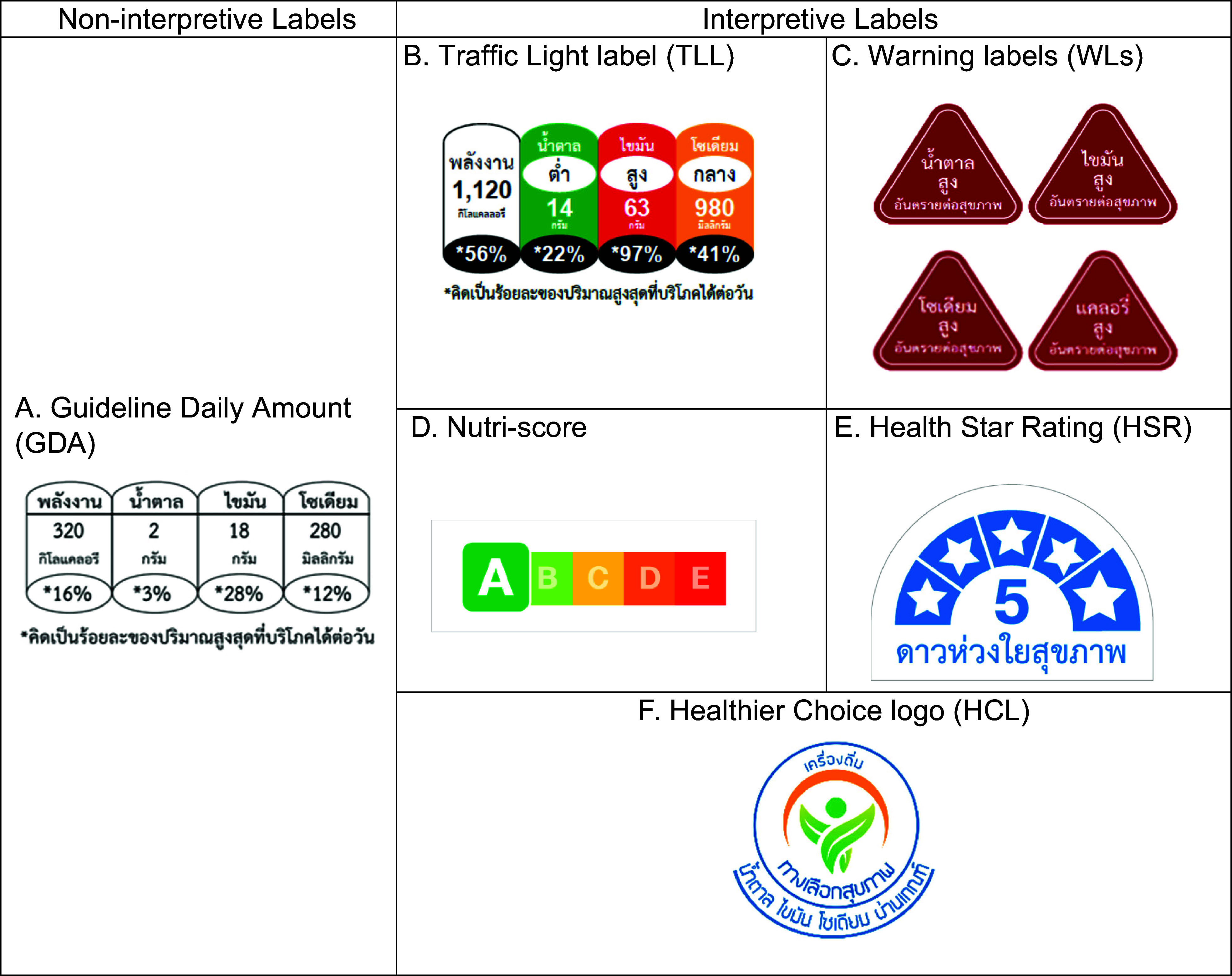
Front-of-pack labelling formats used in the study, categorised as non-interpretive and interpretive labels. English version: (A) Guideline Daily Amount (GDA) (read from left to right): calories 320 kcal, sugar 2 g, fat 18 g, sodium 280 mg. *Calculated as a percentage of the recommended daily intake. (B) Traffic Light label (TLL) (read from left to right): calories 1120 kcal, sugar (low) 14 g, fat (high) 63 g, sodium (medium) 980 mg. *Calculated as a percentage of the recommended daily intake. (C) Warning labels (WLs) (read from left to right): high sugar – health risk, high fat – health risk, high sodium – health risk, high calories – health risk. (E) Health Star Rating (HSR): Health Star care about health. (F) Healthier Choice logo (HCL): beverage, healthier choice, sugar, fat, sodium passed.

While the WHO recommends interpretive FOPL, it encourages countries to conduct consumer testing to understand the impact of FOPL in the specific context^(9)^. Since FOPLs are relatively new, research is still ongoing regarding the most effective types of interpretive labels and their effectiveness in modifying consumers’ purchase decisions towards healthful choices^(3)^. Our research builds on previous FOPL studies in Thailand with additional benefits from a mixed-methods approach to gain comprehensive insights into consumer use of FOPL.

This study aimed to assess Thai consumers’ comprehension, preferences and acceptance of alternative FOPL formats compared with existing systems, generating evidence to support policy recommendations for improving nutrition labelling effectiveness in Thailand. Beyond informing Thai policy, this research contributes to the global evidence base on FOPL effectiveness, particularly for countries implementing or considering transitions from non-interpretive to interpretive labelling systems. The mixed-methods approach and consumer testing framework demonstrated here can be adapted for use in other national contexts.

## Methods

An adapted conceptual framework from Grunert and Wills guided the research design^(18)^, which considers individual factors, environmental factors and FOPL format characteristics about exposure, perception, understanding, liking and use of FOPL.

### Study design

A cross-sectional online survey design was employed to assess consumer understanding, preferences and acceptance of different FOPL formats. Data collection was conducted between February and March 2022 using a structured questionnaire administered via Google Forms. The questionnaire was designed to capture five key domains: (1) demographic characteristics (age, gender, education level, occupation and food purchasing responsibility); (2) dietary behaviour patterns (consumption frequency of instant noodles, snacks, sugar-sweetened beverages and frozen ready-to-eat meals); (3) current use and comprehension of existing FOPL systems in Thailand (GDA labels and HCL); (4) objective understanding of different FOPL; and (5) perceptions and preferences regarding six different FOPL formats.

The FOPL formats evaluated included Thailand’s existing GDA label and HCL, alongside four international formats: WLs use stop-sign symbols to highlight when products are high in nutrients of concern (implemented Latin America such as in Chile, Mexico, Peru, Uruguay)^(19)^; Nutri-Score uses a five-colour scale from A to E to indicate overall nutritional quality (implemented in European countries such as France, Belgium, Germany, Spain)^(20)^; Health Star Rating (HSR) displays 0·5–5 stars based on nutritional profile (implemented in Australia and New Zealand)^(21)^; and Traffic Light labels (TLL) use red, amber and green colours to indicate high, medium or low levels of specific nutrients (implemented in the United Kingdom)^(22)^. These international formats were selected based on their demonstrated effectiveness in other countries and implementation in national food policies^(19,23–26)^.

### Recruitment and procedures

Sample size was determined using Cochran’s formula with the following parameters: *P* = 0·85 (proportion of population who had previously read front-of-package nutrition labels^(17)^), Z = 1·96 (95 % confidence level) and e = 0·05 (acceptable sampling error). The calculated sample size was 196 participants. To compensate for incomplete data, the sample was increased by a factor of 2, resulting in a target sample of 392 participants.

A purposive sampling technique was employed to recruit Thai residents aged 12–70 years who could read and comprehend the Thai language proficiently. Participants were recruited from Bangkok and metropolitan areas through multiple online channels, including community health volunteer networks, student networks from educational institutions and parent associations. The study was promoted as a survey about food packaging and shopping experiences in Thailand without specifically mentioning nutrition labels or health to minimise selection bias towards nutrition-conscious individuals.

### Objective understanding and perceptions and preferences of front-of-pack labels

We tested the objective understanding of FOPL, including the GDA label, WLs, Nutri-Score, HSR and TLL. Participants were asked to select the snack with the highest nutritional quality based on three different variations (healthiest, moderate and least healthy) of each FOPL. Note that HCL was not included in this test due to its lack of variation (e.g. either a product displays a label or does not). We tested these labels on a common packaged snack product (potato chips), using standardised nutrient criteria from established systems. The nutritional content in the GDA label was based on the daily intake recommendations. For WLs, we applied thresholds for sugar, sodium and saturated fat based on the Chilean model^(27)^; for Nutri-Score, we used the criteria from a French study^(28)^; for TLL, we applied UK standards^(22)^; and for HSR, we used the Australian/New Zealand formula^(21)^. Participants were presented with visual examples of each FOPL format to aid their evaluation. Products were presented in randomised orders to prevent order effects. The perceptions and preferences section assessed three main attributes for each FOPL format, including liking (e.g. *‘I like this label’*), attractiveness (e.g. *‘This label is easy to identify’*) and perceived cognitive workload (e.g. *‘This label is too complex to understand’*). Participants rated their agreement with statements using a 5-point Likert scale (strongly disagree to strongly agree). These attributes were validated by Vargas-Meza *et al.* in their Mexican study^(29)^. A pilot study was conducted with thirty-seven participants to assess questionnaire reliability, demonstrating excellent internal consistency for the overall FOPL evaluation questionnaire (*α* = 0·956).

### Qualitative study

Semi-structured interviews were conducted in March 2022. We selected participants through purposive sampling from the online survey. Inclusion criteria were volunteering to be interviewed and having seen the existing GDA label. Sample size was determined using the principle of data saturation, which was reached after twenty-two interviews when no new themes emerged.

Interviews were led by a member of the research team (PaP) who has a background in public health and food and nutrition policy. All interviews were conducted in Thai via Zoom and Line applications, lasting approximately 30–35 min each. A semi-structured interview guide was used, focusing on three main areas: attitudes towards various FOPL formats, the influence of labels on food purchasing decisions and the utilisation of nutritional information in food selection. All interviews were audio-recorded and transcribed verbatim, with participant identities anonymised to protect confidentiality.

### Data analysis

Quantitative data analysis was performed using STATA version 16. Descriptive statistics were used to summarise participant characteristics and responses. Chi-square tests were employed to compare perceptions and preferences across different groups, while multiple logistic regression was used to analyse the relationship between label perceptions and demographic factors.

Our sample of 410 participants provided adequate power for the regression analyses conducted. The sample size with eight predictor variables (e.g. sex, age groups, educational level, etc.) in the logistic regression model exceeded recommended events-per-variable thresholds, based on the literature^(30–32)^. All regression assumptions, including the absence of multicollinearity and adequate model fit, were verified.

Thematic analysis was employed to analyse the qualitative data. This process involved transcription, double coding and categorisation of data to identify recurring themes and patterns. The analysis was guided by the research questions and conceptual framework of the study. Qualitative analysis was performed independently by two researchers (PaP and PhP), using Microsoft Excel to facilitate double coding, categorisation and inter-rater reliability assessment. The research team comprised public health nutritionists and policy researchers with expertise in food labelling.

## Results

### Findings from the survey

#### Study characteristics

The survey sample (*n* 410) was predominantly female (75·4 %), with a mean age of 31 years (range: 12–78 years). The age distribution was skewed towards adults, with 31·6 % aged 12–18 years, 46·8 % aged 19–50 years and 21·2 % aged 51 years and above. Educational attainment varied, with 40·2 % holding a bachelor’s degree or higher. Occupations were diverse, with students comprising the largest group (38·5 %), followed by company workers (14·8 %) and civil servants (13·6 %). Just over half (51·0 %) of the respondents were primarily responsible for buying food for their households. Importantly, more than 68·3 % of respondents considered nutrition as a factor in their food purchasing decisions, and over 50 % reported buying food from convenience stores (Table 1).

**Table 1.** Study characteristics (*n* 410)

Variables		*n*	%
Gender	Female	309	75·4
	Male	101	24·6
Age groups (mean = 31 years)	12–18 years	131	31·6
	19–50 years	192	46·8
	51 years and more	87	21·2
Educational level	Primary	29	7·1
	Secondary	111	27·1
	High school	87	21·2
	Diploma	18	4·4
	Bachelor’s and higher	165	40·2
Occupation	Student	158	38·5
	Company workers	61	14·8
	Civil servant/working for government sector	56	13·6
	Own business	39	9·5
	General employed	33	8·0
	Farmer	20	4·9
	Unemployed/retired	43	10·5
Responsible for buying food	Yes	209	51·0
	No	201	49·0
Primary place to buy foods	Fresh market	83	20·2
	Local grocery store	25	6·1
	Convenience store	214	52·2
	Supermarket/hypermarket	79	19·3
	Online	8	2·0
	Others	1	0·2
Prioritise nutritional value or nutrients when purchasing food	Yes	280	68·3
	No	130	31·7

Dietary behaviour analysis revealed that consumption patterns differed across age groups. Remarkably, 19·8 % of the 12–18 age group consumed snacks daily, while only 8·9 % of those aged 19–50 and 0 % of those over 50 did so. Additionally, over 70 % of adults aged 19–50 consumed sugar-sweetened beverages and frozen ready-to-eat meals at least occasionally, compared with much lower rates in other age groups. Instant noodle consumption was high across all age groups, with more than 90 % consuming them sometimes (see online supplementary material, Supplemental Table S1).

#### Use, awareness and understanding of front-of-pack labels

##### Use and awareness of existing front-of-pack labels in Thailand

Regarding the use and awareness of the GDA and HCL, the findings showed that 95·4 % of respondents had seen the GDA label, but only 23·9 % reported reading it every time they purchased food. The main reasons for not reading GDA were familiarity with the products (55·9 %) and prioritising price (53·4 %). While 69·8 % of respondents self-reported that they understood the GDA label’s meaning, this represents perceived comprehension rather than demonstrated ability to interpret the information correctly, which was also tested (results below). Similarly, for the HCL, 82·4 % reported having seen it, and 77·8 % self-reported that they understood its meaning, though actual comprehension was not tested, as noted earlier.

##### Understanding of different front-of-pack labels

**Figure 2.**
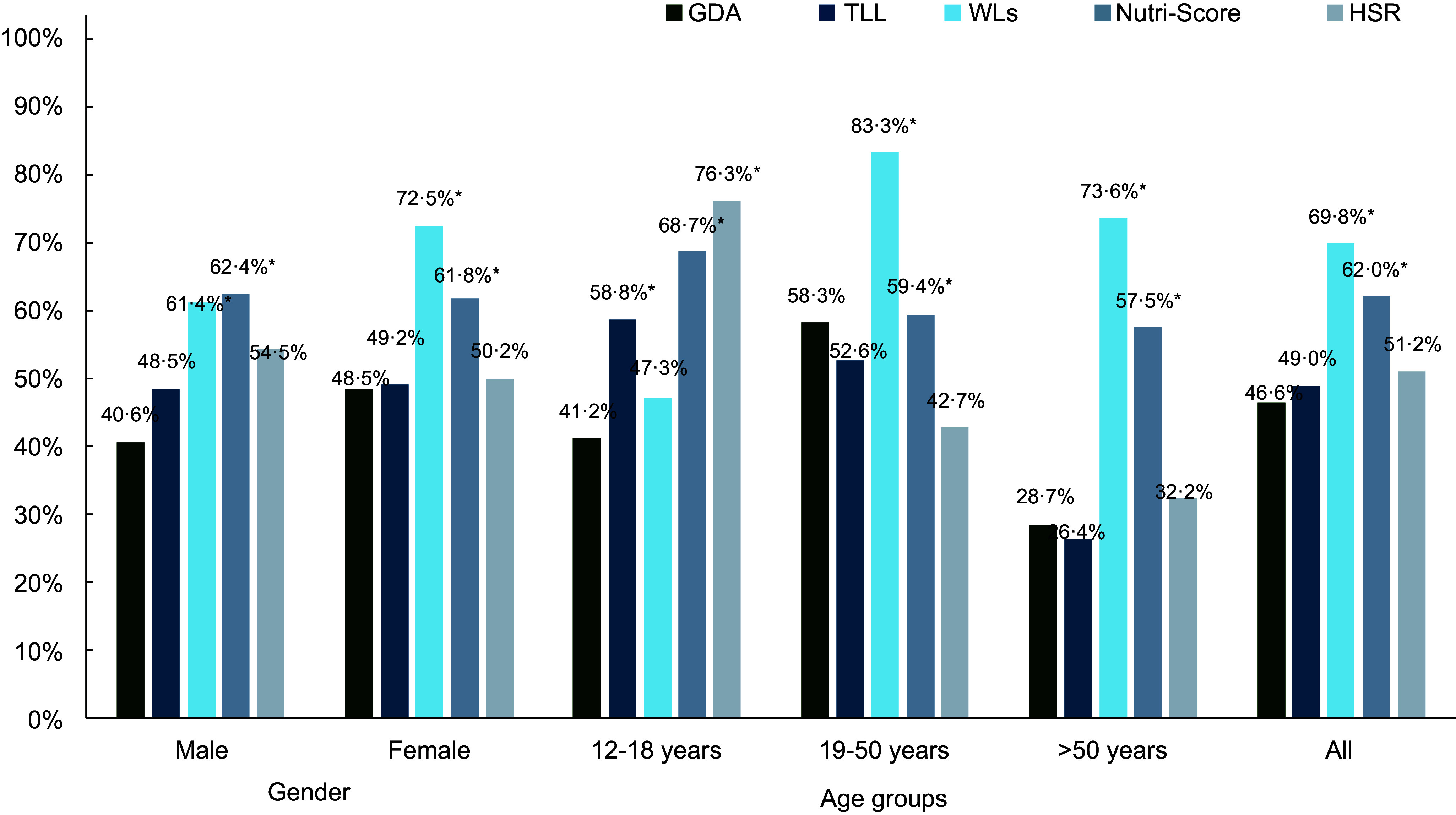
Proportion of participants correctly identifying the product with the highest nutritional quality using different types of labels by gender and age groups (*n* 410). *Indicate statistically significant differences (*P* < 0·05) compared with the GDA label as the reference group. GDA, Guideline Daily Amount; TLL, Traffic Light label; WLs, warning labels; HSR, Health Star Rating.

Next, consumer understanding of labels was tested by asking participants to identify the healthiest item among three snack food options. The WLs and Nutri-Score consistently showed higher proportions of correct responses in product identification compared with other label formats, with statistically significant differences compared with the GDA label as a reference (*P*-value < 0·05). The performance gap between these two labels and the current GDA label remained consistent across all demographic subgroups, with the GDA label showing consistently lower correct response rates (ranging from 28·7 % to 58·3 %) (Figure 2).

##### Factors influencing understanding of front-of-pack labels

Demographic factors showed distinct patterns of influence across different label formats (see online supplementary material, Supplemental Table S2). Age emerged as the most consistent predictor of label understanding, with significant effects observed across multiple formats. WLs were better understood by older age groups, with both the 19–50 years (adjusted OR: 3·33, 95 % CI: 1·16, 9·58) and over 50 years groups (adjusted OR: 4·88, 95 % CI: 1·49, 15·98) showing significantly higher understanding compared with the 12–18 age group. In contrast, younger participants (12–18 years) demonstrated better comprehension of the TLL and HSR, with older age groups showing significantly lower odds of correct interpretation (adjusted OR: 0·11, 95 % CI: 0·04, 0·30 and OR: 0·07, 95 % CI: 0·02, 0·22, respectively). Education level primarily affected understanding of the TLL, with those holding bachelor’s degrees showing notably higher comprehension (adjusted OR: 9·34, 95 % CI: 2·57, 33·88). Geographic and economic factors were most relevant for the GDA label, with participants outside municipal areas showing lower understanding (adjusted OR: 0·55, 95 % CI: 0·32, 0·93) and those with higher income (> 25 000 baht) demonstrating better comprehension (adjusted OR: 3·39, 95 % CI: 1·21, 9·50). Furthermore, gender, chronic disease status and BMI showed no significant association with label understanding across any format.

#### Perceptions and preferences among various front-of-pack labels

The HCL received the highest levels of agreement across multiple perceptual attributes, particularly in packaging inclusion preference (59·8 %), visibility (58·5 %) and attractiveness (57·3 %) (Table 2). The HSR and TLL also performed strongly, with HSR achieving high ratings for visual appeal (56·6 %) and inclusion preference (56·1 %), while TLL was valued for providing desired information (56·5 %) and reliable information (56·6 %). WLs, despite demonstrating effectiveness in guiding healthier choices elsewhere in the study, received relatively lower consumer preference ratings across most attributes, with scores ranging from 44·9 % to 52·2 %. It is worth noting that, despite having the lowest attractiveness rating (40·2 %), the GDA label maintained comparable scores for information reliability (56·3 %), suggesting that consumers trust its accuracy despite its less appealing visual design.

**Table 2.** Proportion of participants who strongly agreed or agreed to the statements evaluating perceptions and preferences among various front-of-pack labels (*n* 410)

Statements	GDA (reference)		HCL			TLL			WLs			Nutri-Score			HSR		
	*n*	%	*n*	%	*P*-value	*n*	%	*P*-value	*n*	%	*P*-value	*n*	%	*P*-value	*n*	%	*P*-value
I like this label	176	42·9	232	56·6	< 0·0001***	211	51·5	< 0·0001***	184	44·9	0·4752	185	45·1	0·4351	216	52·7	< 0·0001***
I want to see this label on all food packaging	206	50·2	245	59·8	< 0·0001***	228	55·6	0·0013**	202	49·3	0·7465	207	50·5	1·0000	230	56·1	0·0097***
This label is attractive	165	40·2	235	57·3	< 0·0001***	225	54·9	< 0·0001***	193	47·1	0·0034**	212	51·7	< 0·0001***	232	56·6	< 0·0001***
This label provides the desired information	224	54·6	221	53·9	0·8126	230	56·5	0·1628	203	49·5	0·0170*	195	47·6	0·0022***	211	51·5	0·1654
This label is easy to notice	181	44·1	240	58·5	< 0·0001***	230	56·1	< 0·0001***	214	52·2	0·0003***	213	52·0	0·0007***	230	56·1	< 0·0001***
This label provides reliable/useful information	231	56·3	238	57·5	0·7948	232	56·6	1·0000	207	50·5	0·0022***	196	47·8	< 0·0001***	212	51·7	0·0319*

GDA, Guideline Daily Amount; HCL, Healthier Choice logo; TLL, Traffic Light label; WLs, warning labels; HSR, Health Star Rating.

*P ≤ 0·05, **P ≤ 0·01, ***P ≤ 0·001.

### Qualitative findings

Based on semi-structured interviews with twenty-two participants, three key themes emerged regarding FOPL use and understanding:

#### Current label awareness and comprehension

Participants demonstrated complex engagement patterns with Thailand’s existing FOPL labels. While awareness of both the GDA label and HCL was high, understanding varied substantially across label types. The GDA label format posed significant comprehension challenges for participants across education levels, with many struggling to interpret its numerical presentation of nutritional information. This difficulty was clearly expressed by multiple participants, for example,

‘I don’t understand the percentage or numbers in the GDA label’ (Female, 36, bachelor)

and

‘I ever saw it (GDA), but I just don’t get what it means’. (Female, 53, primary).

In contrast, participants consistently reported better engagement with the HCL, primarily attributing this to its use of colour as a visual cue for healthfulness.

#### Barriers to label use

Several significant barriers emerged that hindered effective label use among Thai consumers. Time constraints during shopping emerged as a critical barrier, particularly among urban residents who frequently shopped at convenience stores and supermarkets where quick purchasing decisions were common, as exemplified by one participant who noted that:

‘I don’t really notice them because I’m usually in a hurry when shopping’. (Female, 55, primary).

The physical design of current labels presented another substantial challenge, with participants consistently highlighting issues with font size that made quick reading difficult, while the lack of intuitive colour-coding further complicated rapid comprehension during shopping trips, as reflected in participants’ suggestions:

‘The GDA is not attractive for me because of the design is not interesting and the letters are too small’ (Male, 56, high school)

and

‘I just would like to see more colorful label than what we have nowadays, I think this can help people to understand the label’. (Male, 30, high school).

Beyond these practical constraints, a fundamental barrier lay in consumers’ difficulty interpreting and contextualising nutritional information meaningfully for their health needs, particularly when confronted with percentage-based information that lacked clear health implications.

‘When they say this food contains 15 % fat, I just don’t know if this amount is okay for my body or not’. (Female, 36, bachelor).

The final significant barrier stemmed from deeply ingrained shopping habits, where established purchasing patterns and brand loyalty often superseded label consideration in the decision-making process.

‘I’ve always bought that product, so I just don’t read the label’. (Male, 22, bachelor).

#### Consumer preferences and suggestions for improvement

The first and most prominent was an overwhelming desire for colour-coded labelling systems, particularly those that mimicked the universally understood TLL scheme. This preference stemmed from participants’ belief that colour-coding would facilitate quick, intuitive decision-making during shopping, with many participants specifically highlighting how green signalled safety and health in their minds. As articulated by one participant:

‘When I see green, I feel safe. It would be good to have green, yellow, red like traffic lights. It would be easy to understand’. (Female, 49, primary).

The second major theme centred on the recognised need for comprehensive public education about label use and interpretation. Participants emphasised that while improved label design was crucial, it needed to be accompanied by widespread public education campaigns to ensure effective utilisation across all population segments.

‘Advertising or education campaign is needed to help people understand the label, because someone know what the label is but someone doesn’t’. (Female, 31, bachelor).

## Discussion

This mixed-methods study revealed complex patterns in the acceptability, perception, preference, understanding and intention to use different types of FOPL in Thailand. While we found high awareness of existing labels (GDA label: 95·4 %, HCL: 82·4 %), this awareness did not translate into meaningful engagement, with participants expressing difficulties in interpreting numerical information, particularly for the GDA label format. The findings highlighted a strong preference for colour-coded systems, with participants specifically noting that ‘green colour signals safety’ and expressing a desire for TLL-style indicators for easier interpretation. However, quantitative data revealed that interpretive formats, particularly WLs, demonstrated superior effectiveness in helping consumers identify healthier products across demographic groups – the key reason for requiring FOPL.

This contrast between stated preferences (favouring symbolic and colour-coded labels like TLL and HCL) and measured effectiveness (where WLs performed best) represents an important finding for policy development. It suggests that consumer preference alone may not be sufficient to guide FOPL or food and nutrition policy decisions; effectiveness in promoting healthier choices must be a primary consideration^(3,33)^. This divergence also indicates the need for further investigation into how different label formats influence both consumer perception and decision-making behaviour, as well as the potential role of public education campaigns in enhancing acceptance of evidence-based label designs^(34,35)^.

The findings that an interpretive label, particularly the WLs, was the most effective in guiding consumers to healthier products align with WHO recommendations and global research^(3,9)^. Interpretive labels that quickly provide consumers with clear information about the nutritional quality of the product are the most effective types of labels and are widely used globally^(9)^. In this study, the two interpretive labels that provide consumers with information that facilitates distinguishing between products with different nutritional profiles are the Nutri-Score and WLs designs. The Nutri-Score is already in use in several European countries such as France and Germany, and WLs are implemented in Latin America, including Brazil; both systems have demonstrated an impact^(36,37)^. Consistent with findings here, some studies suggest that WLs are particularly effective at providing critical information about foods that may be perceived as healthy but contain high levels of nutrients of concern^(3)^. Notably, interpretive systems like these are more likely than non-interpretive systems to prompt industry reformulation^(10)^.

The GDA label received the lowest score in use and understanding. Our findings regarding the limited effectiveness of the current GDA label in Thailand are consistent with previous research^(17)^, which demonstrated consistent minimal impact of the GDA label over time on helping consumers choose healthier products. Furthermore, our results are consistent with studies from other countries showing that the GDA label is less effective in improving consumers’ perception and understanding of healthy and unhealthy foods when compared with other interpretive labels such as WLs^(38,39)^. This suggests that replacing the GDA label with a more interpretive label, such as a WLs, could significantly enhance consumers’ ability to make informed nutritional decisions, as recommended by the WHO^(7)^. The qualitative data from our study further support this notion, with participants expressing difficulties in understanding percentage values and contextualising nutritional information presented in the GDA label format.

The identified barriers to FOPL use, including time constraints during shopping and small font sizes, highlight the importance of developing labels that can be quickly and easily interpreted. These findings are particularly relevant in Thailand’s rapidly transforming food landscape, characterised by increasing urbanisation and a shift towards convenience store shopping. This transition is marked by greater accessibility to packaged foods and changing consumer behaviours^(40,41)^, particularly in urban areas^(42)^, where time constraints influence shopping patterns. The importance of a FOPL design that can be interpreted quickly, given these time constraints during shopping, is a key factor for consideration when developing a policy. Evidence from other countries has consistently found that WLs are among the easiest and quickest to interpret correctly, making them particularly well-suited for Thailand’s convenience-oriented shopping environment. As such, implementing an effective FOPL system could serve as a crucial tool to promote healthier dietary choices and address the rising burden of diet-related non-communicable diseases in the country.

### Policy implications

This study demonstrates the need for clearer, more intuitive labelling. There is a strong case for reforming the current GDA label, given its limited utilisation and documented comprehension difficulties. The superior performance of WLs across demographic groups, particularly among adults, suggests that implementing interpretive WLs could significantly enhance consumer understanding and healthy food choice behaviours.

A comprehensive public education campaign should accompany any FOPL policy changes. This recommendation is directly supported by our qualitative findings, where participants explicitly requested better educational support for label interpretation. Such campaigns should include practical guidance on interpreting nutritional information and its relevance to health decisions, while also addressing the habitual purchasing behaviours identified as barriers to label use. Special attention should be paid to consumers outside municipal areas, who demonstrated lower comprehension of current labels, and to addressing price sensitivity as a barrier to healthy food choices.

To ensure long-term effectiveness, we recommend establishing robust monitoring and evaluation systems that assess both objective understanding and real-world usage patterns across different demographic groups. This approach would allow for continuous refinement of both the labelling system and associated educational initiatives, ensuring that the FOPL system effectively serves its intended purpose of facilitating healthier food choices among Thai consumers. These recommendations align with global best practices while addressing the specific challenges and needs identified in the Thai context.

### Strengths and limitations

This study has several notable strengths. First, the mixed-methods design provided complementary insights into FOPL effectiveness, combining quantitative measures of label comprehension with rich qualitative data about user experiences and preferences. The inclusion of both urban and rural participants, along with diverse age groups (12–78 years) and educational backgrounds, enhanced the generalisability of our findings. Additionally, our study examined multiple FOPL formats simultaneously, including both existing Thai labels and international label formats, which have evidence to support their effectiveness, allowing for direct comparisons. The inclusion of objective understanding tests, rather than relying solely on self-reported comprehension, provided more reliable evidence of label effectiveness.

However, several limitations should be considered. The use of convenience sampling through online channels from community health volunteer networks, educational institutions and parent associations may have introduced selection bias towards individuals with higher health literacy, greater interest in nutrition and better access to digital technology. This may have resulted in overestimation of label comprehension and underrepresentation of vulnerable populations with lower digital literacy or limited health engagement. Additionally, the predominantly female sample and high proportion of participants with bachelor’s degrees or higher may not fully represent the population proportion in Thailand. Furthermore, the cross-sectional design captures only a snapshot of FOPL understanding and preferences, without examining how comprehension and usage patterns might change over time. The testing of label understanding was conducted in a controlled setting, which may not fully reflect real-world shopping conditions where time constraints, competing priorities and environmental factors influence label use. Additionally, while the study included adolescents (12–18 years), their responses were not analysed separately from adults, which might mask age-specific patterns in label comprehension and preferences. Finally, this research does not consider how the food industry might respond to different types of labels through reformulation, which would influence the overall health impact.

Future research could address these limitations through longitudinal studies examining FOPL effectiveness over time, inclusion of more representative samples and real-world testing of label use in actual shopping environments. Studies specifically focused on underrepresented groups, such as elderly populations and those with lower educational attainment, would also be valuable for developing targeted interventions.

### Conclusion

This study provides evidence regarding FOPL effectiveness in Thailand. While the GDA label and HCL achieve high awareness, they face significant challenges in facilitating informed food choices. The superior performance of WLs in guiding product selection suggests a clear direction for policy reform, though the disconnect between measured effectiveness and stated preferences highlights the need for a complementary education campaign.

The findings have important policy implications, supporting a transition from the current GDA system to more interpretive formats, accompanied by targeted educational campaigns that address diverse demographic needs. Future research should examine real-world implementation and long-term impacts on consumer behaviour and industry practices. These findings provide valuable evidence for developing effective FOPL systems that can contribute to addressing Thailand’s diet-related non-communicable diseases and inform similar policy initiatives globally.

## Supporting information

Phonsuk et al. supplementary material Phonsuk et al. supplementary material

## Supplementary material

For supplementary material accompanying this paper, visit https://doi.org/10.1017/S1368980026101852.
