# Supplementary material for: Consumer understanding, preferences and acceptance of front-of-pack labels in Thailand: foundational evidence for policy development
Source: Public Health Nutr. 2026 Jan 22;29(1):e45. doi: 10.1017/S1368980026101852 (PMC13087988; doi:10.1017/S1368980026101852)
Supplement: Phonsuk et al. supplementary material [file S1368980026101852sup001.docx]

**Supplementary materials**

**Supplementary Table S1: Dietary behavior among participants, according to age groups (N=410)**

| **Food types** | **Frequency** | **Age groups** | | | **Total**  n (%) |
| --- | --- | --- | --- | --- | --- |
|  |  | **12-18 yrs**  n (%) | **19-50 yrs**  n (%) | **>50 yrs**  n (%) |  |
| **Instant noodle** | No | 10 (7.6) | 14 (7.3) | 10 (11.5) | 34 (8.3) |
|  | Eat sometime* | 118 (90.1) | 176 (91.7) | 77 (88.5) | 371 (90.5) |
|  | Eat everyday | 3 (2.3) | 2 (1.0) | 0 (0.0) | 5 (1.2) |
| **Snack** | No | 4 (3.1) | 4 (2.1) | 17 (19.5) | 25 (6.1) |
|  | Eat sometime* | 101 (77.1) | 171 (89.1) | 70 (80.5) | 342 (83.4) |
|  | Eat everyday | 26 (19.8) | 17 (8.9) | 0 (0.0) | 43 (10.5) |
| **Frozen ready-to-eat** | No | 42 (32.1) | 42 (21.9) | 48 (55.2) | 132 (32.2) |
|  | Eat sometime* | 85 (64.9) | 148 (77.1) | 39 (44.8) | 272 (66.3) |
|  | Eat everyday | 4 (3.1) | 2 (1.0) | 0 (0.0) | 6 (1.5) |
| **Sugar-sweetened beverages** | No | 9 (6.9) | 7 (3.7) | 12 (13.8) | 28 (6.8) |
|  | Eat sometime* | 105 (80.2) | 146 (76.0) | 72 (82.8) | 323 (78.8) |
|  | Eat everyday | 17 (13.0) | 39 (20.3) | 3 (3.4) | 59 (14.4) |

***Consume more than 1 day in a month**

Supplementary Table S2: Factors influencing the effectiveness of different FOPL formats

| **Variables** | **GDA**  **(n = 394)** | | | **TLL  (n = 389)** | | | **WLs**  **(n = 392)** | | | **Nutri-score**  **(n = 401)** | | | **HSR**  **(n =398)** | | |  |
| --- | --- | --- | --- | --- | --- | --- | --- | --- | --- | --- | --- | --- | --- | --- | --- | --- |
|  | **Adj OR** | **95%Ci** | **p-value** | **Adj OR** | **95%Ci** | **p-value** | **Adj OR** | **95%Ci** | **p-value** | **Adj OR** | **95%Ci** | **p-value** | **Adj OR** | **95%Ci** | **p-value** | |
| **Sex** |  |  |  |  |  |  |  |  |  |  |  |  |  |  |  | |
| Male | 1.00 |  |  | 1.00 |  |  | 1.00 |  |  | 1.00 |  |  | 1.00 |  |  | |
| Female | 1.33 | 0.78 -2.27 | 0.293 | 1.32 | 0.78 - 2.26 | 0.301 | 0.99 | 0.54 - 1.82 | 0.975 | 1.14 | 0.67 - 1.94 | 0.626 | 1.60 | 0.91 - 2.80 | 0.101 | |
| **Age groups** |  |  |  |  |  |  |  |  |  |  |  |  |  |  |  | |
| 12-18 yrs | 1.00 |  |  | 1.00 |  |  | 1.00 |  |  | 1.00 |  |  | 1.00 |  |  | |
| 19-50 yrs | 1.01 | 0.41 - 2.49 | 0.982 | **0.11** | **0.04 - 0.30** | **<0.001***** | **3.33** | **1.16 - 9.58** | **0.026*** | **0.40** | **0.16 - 0.99** | **0.047*** | **0.09** | **0.03 - 0.25** | **<0.001***** | |
| 51 yrs and more | 0.43 | 0.16 - 1.17 | 0.098 | **0.07** | **0.02 - 0.22** | **<0.001***** | **4.88** | **1.49 - 15.98** | **0.009**** | **0.43** | 0.16 - 1.15 | 0.094 | **0.07** | **0.02 - 0.20** | **<0.001***** | |
| **Educational level** |  |  |  |  |  |  |  |  |  |  |  |  |  |  |  | |
| Primary | 1.00 |  |  | 1.00 |  |  | 1.00 |  |  | 1.00 |  |  | 1.00 |  |  | |
| Secondary | 1.37 | 0.40 - 4.70 | 0.618 | 0.61 | 0.16 - 2.37 | 0.477 | 0.91 | 0.29 - 2.85 | 0.873 | 0.46 | 0.15 - 1.37 | 0.162 | 0.45 | 0.14 - 1.39 | 0.164 | |
| High school | 2.83 | 0.89 - 8.98 | 0.078 | 1.98 | 0.59 - 6.65 | 0.270 | 2.33 | 0.79 - 6.92 | 0.127 | 0.75 | 0.27 - 2.08 | 0.583 | 0.60 | 0.22 - 1.63 | 0.316 | |
| Diploma | 1.23 | 0.27 - 5.66 | 0.793 | 3.30 | 0.76 - 4.44 | 0.112 | 4.17 | 0.71 - 24.42 | 0.113 | **0.22** | **0.06 - 0.82** | **0.024*** | 1.19 | 0.33 - 4.34 | 0.787 | |
| Bachelor and higher | 3.06 | 0.87 - 10.67 | 0.080 | **9.34** | **2.57 - 33.88** | **<0.001***** | 2.20 | 0.62 - 7.84 | 0.225 | 0.71 | 0.23 - 2.19 | 0.553 | 0.82 | 0.27 - 2.44 | 0.716 | |
| **Living area** |  |  |  |  |  |  |  |  |  |  |  |  |  |  |  | |
| municipal area | 1.00 |  |  | 1.00 |  |  | 1.00 |  |  | 1.00 |  |  | 1.00 |  |  | |
| Non municipal area | **0.55** | **0.32 - 0.93** | **0.027*** | 0.90 | 0.52 - 1.53 | 0.688 | 1.16 | 0.62 - 2.16 | 0.638 | 0.82 | 0.49 - 1.35 | 0.432 | 1.21 | 0.72 - 2.04 | 0.470 | |
| **Average income (baht/month)** |  |  |  |  |  |  |  |  |  |  |  |  |  |  |  | |
| None | 1.00 |  |  | 1.00 |  |  | 1.00 |  |  | 1.00 |  |  | 1.00 |  |  | |
| <7,000 | 1.12 | 0.60 - 2.13 | 0.720 | 1.15 | 0.59 - 2.23 | 0.681 | 0.50 | 0.25 - 1.02 | 0.056 | 0.64 | 0.33 - 1.24 | 0.185 | 0.73 | 0.36 - 1.49 | 0.384 | |
| 7,001-15,000 | 1.11 | 0.50 - 2.46 | 0.792 | 1.02 | 0.45 - 2.32 | 0.963 | **0.37** | **0.14 - 0.94** | **0.038*** | 0.92 | 0.41 - 2.05 | 0.840 | 0.81 | 0.35 - 1.85 | 0.613 | |
| 15,001-25,000 | 1.38 | 0.55 - 3.49 | 0.496 | 0.80 | 0.31 - 2.06 | 0.638 | 2.46 | 0.55 - 11.03 | 0.238 | 1.28 | 0.50 - 3.29 | 0.610 | 1.10 | 0.43 - 2.79 | 0.847 | |
| >25,000 | **3.39** | **1.21 - 9.50** | **0.020*** | 0.99 | 0.37 - 2.60 | 0.976 | 1.26 | 0.34 - 4.76 | 0.731 | 0.75 | 0.29 - 1.94 | 0.548 | 0.85 | 0.30 - 2.22 | 0.734 | |
| **Having chronic disease** |  |  |  |  |  |  |  |  |  |  |  |  |  |  |  | |
| No | 1.00 |  |  | 1.00 |  |  | 1.00 |  |  | 1.00 |  |  | 1.00 |  |  | |
| Yes | 1.24 | 0.69 - 2.25 | 0.469 | 1.49 | 0.80 - 2.79 | 0.210 | 1.04 | 0.53 - 2.02 | 0.913 | 1.13 | 0.64 - 2.02 | 0.674 | 1.13 | 0.63 - 2.04 | 0.680 | |
| **BMI** |  |  |  |  |  |  |  |  |  |  |  |  |  |  |  | |
| <18.5 | 1.00 |  |  | 1.00 |  |  | 1.00 |  |  | 1.00 |  |  | 1.00 |  |  | |
| 18.5-22.9 | 1.45 | 0.78 - 2.73 | 0.242 | 0.58 | 0.30 - 1.11 | 0.100 | 1.24 | 0.62 - 2.48 | 0.548 | 1.43 | 0.76 - 2.70 | 0.265 | 1.03 | 0.52 - 2.04 | 0.930 | |
| >23 | 1.68 | 0.89 - 3.17 | 0.107 | 0.89 | 0.45 - 1.74 | 0.726 | 1.20 | 0.59 - 2.43 | 0.621 | 1.69 | 0.89 - 3.19 | 0.107 | 1.14 | 0.58 - 2.26 | 0.699 | |

**Note:** ∗p ≤ 0.05, ∗∗p ≤ 0.01, ∗∗∗p ≤ 0.001
